# Does lumbar lordosis minus thoracic kyphosis predict the clinical outcome of patients with adult degenerative scoliosis?

**DOI:** 10.1186/s13018-019-1339-y

**Published:** 2019-09-03

**Authors:** Siyu Zhou, Wei Li, Tong Su, Chengbo Du, Wei Wang, Fei Xu, Zhuoran Sun, Weishi Li

**Affiliations:** 1 Department of Orthopaedic, Peking University Third Hospital, No. 49 North Garden Road, Haidian District, Beijing, 100191 China; 2 Peking University Health Science Center, No. 38 Xueyuan Road, Haidian District, Beijing, 100191 China

**Keywords:** Corrective surgery, Adult degenerative scoliosis, Adult spinal deformity, Sagittal balance, Lumbar lordosis, Thoracic kyphosis

## Abstract

**Purpose:**

To evaluate the predictive effect of lumbar lordosis minus thoracic kyphosis (LL-TK) in the surgical outcome of adult degenerative scoliosis (ADS) patients and explore the optimum target base on it.

**Methods:**

The preoperative and postoperative data including radiographic image and functional evaluation (Visual Analog Scale, VAS; Oswestry Disability Index, ODI; Japanese Orthopaedic Association, JOA) of 130 patients with ADS who underwent corrective surgery was retrospectively reviewed. The relationship between sagittal parameters and surgical outcome was assessed by using the Pearson correlation analysis. Receiver operating characteristic (ROC) curve was used to define the optimum cutoff value of LL-TK. Patients were divided into two groups based on LL-TK to compare the preoperative and postoperative status.

**Results:**

LL-TK assessed soon after surgery strongly correlated with health-related quality of life (HRQOL) and sagittal vertical axis (SVA) at last follow-up. The cutoff value of LL-TK was set at 10° to determine a good clinical outcome (ODI < 20) and sagittal balance (SVA < 50 mm). Patients with LL-TK > 10° presented significantly better postoperative VAS, ODI, JOA, and SVA than patients with LL-TK < 10°.

**Conclusion:**

LL-TK could effectively predict postoperative HRQOL and sagittal balance for patients with ADS. Patients with LL-TK > 10° showed a better clinical outcome and sagittal balance, so LL-TK > 10° could be the optimum corrective target for these patients.

## Background

Owing to the degeneration of spinal motion segments, adult degenerative scoliosis (ADS) was a common type of adult spinal deformity (ASD) without history of adolescent idiopathic scoliosis (AIS) [1]. This kind of deformity could be found in approximately 30% of elderly people [2]. ADS could manifest various symptoms including low back pain, radiating pain in lower extremities, neurological deficits, and claudication [3], which significantly impacts patient’s health-related quality of life (HRQOL) [4]. Treatment of adult degenerative scoliosis remained a challenge for spine surgeons. Conservative therapy was initially recommended, but for patients with persistent symptoms, surgical treatment was needed.

In these patients, the key procedure of surgery was to achieve an ideal correction of deformity, especially on sagittal plane, since sagittal balance has been proven to be most closely associated with surgical outcome [5–8]. Schwab et al. proposed a classification for patients with ASD based on three critical sagittal parameters including sagittal vertical axis (SVA), pelvic tilt (PT), and pelvic incidence minus lumbar lordosis (PI-LL), and they pointed out that corrective surgery should reach the objectives of SVA less than 50 mm, PT less than 25°, and PI-LL within 10° [9, 10], which had been widely used in clinical practice.

Apart from these classical sagittal modifiers, another study demonstrated that lumbar lordosis minus thoracic kyphosis (LL-TK) could be a good predictor for sagittal balance in Chinese elderly people [11], since LL-TK reflected the regional compensatory mechanism and significantly associated with SVA. Realizing the importance of this regional predictor, we speculated that LL-TK could also be a good parameter to guide the correction surgery for patients with ADS, which has not been addressed by the previous studies.

Thus, this study was aimed to evaluate the predictive effect of LL-TK on the surgical outcome of patients with ADS and define the suitable correction goal based on this parameter.

## Methods

### Study design and inclusion criteria

This was a single-institution retrospective study approved by the relevant institutional Ethics Committee. A total of 130 patients with ASD treated with corrective surgery in our hospital from January 2010 to December 2016 were included in this study. Visual Analog Scale (VAS), the Oswestry Disability Index (ODI), and Japanese Orthopaedic Association (JOA) Scores were used to assess HRQOL. The inclusion criteria were as follows.

1. Age > 50 years old at the time of surgery
2. Cobb angle > 10° and located in the lumbar spine
3. Long instrument with fusion ≥ 3 vertebral segments
4. Without history of scoliosis up to adolescence
5. Without history of former spinal surgery
6. Without history of neuromuscular diseases
7. With at least 2 years follow-up
8. Had complete preoperative and postoperative data

### Radiographic data

**Fig. 1:**
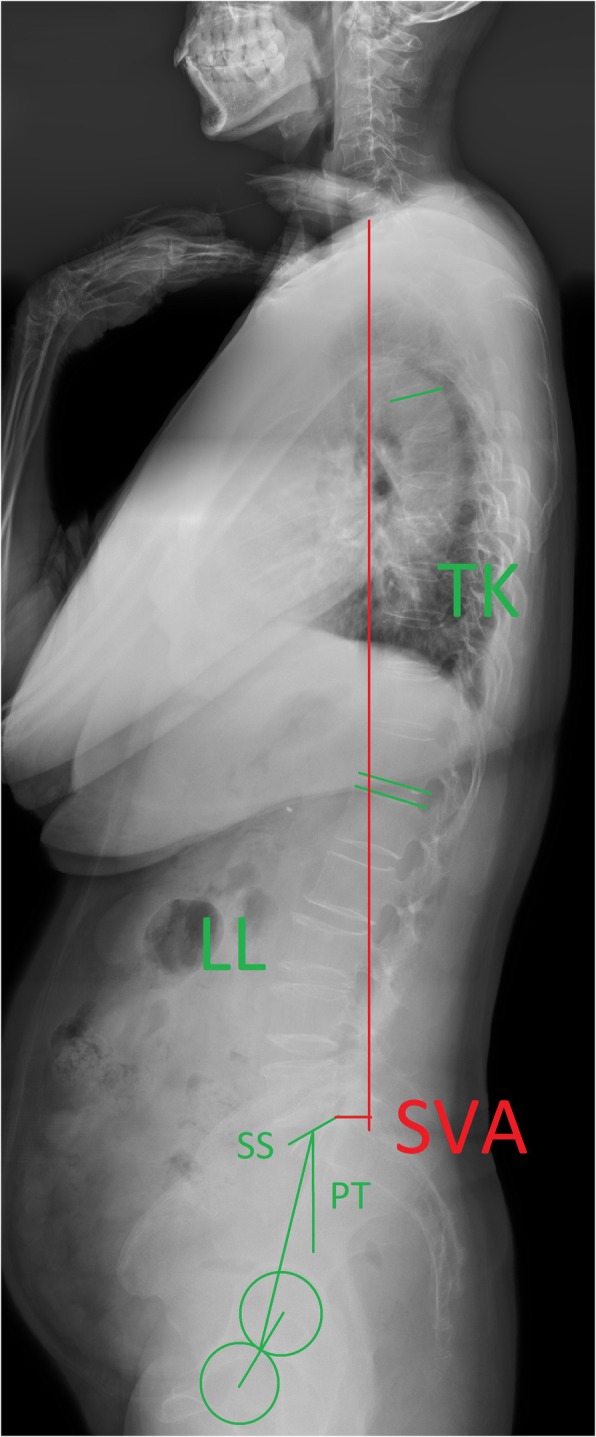
The measurements of sagittal parameters

The radiographic assessments were analyzed by standing posteroanterior and lateral whole spine X-ray preoperatively and postoperatively. The parameters including SVA, lumbar lordosis (LL), thoracic kyphosis (TK), pelvic incidence (PI), pelvic tilt (PT), sacral slope (SS), and Cobb angle of the curves were measured by two experienced orthopedic surgeons who were not otherwise involved in this study, and the average of their results was recorded (Fig. 1).

### Statistical analysis

SPSS version 19.0 (SPSS Inc., Chicago, IL, USA) was used to analyze the collected data. The preoperative and postoperative sagittal and coronal parameters were compared by using paired sample *t* test. Pearson correlation analysis was used to assess the relationship between HRQOL and sagittal parameters. We used receiver operating characteristic (ROC) curve to find out the most optimum cut-off point of which presented the largest Youden index. The comparison in sagittal alignment and clinical outcome between different groups was performed by using independent sample *t* test. *χ*^2^ test was done to compare categorical data in different groups. Statistical significance was set at *P* value < 0.05.

## Result

### General data

There were 29 males and 101 females in the patients’ group. The average age of patients was 63.6 ± 6.1 years. The mean time of follow-up was 3.2 ± 1.4 years. The average segment of fusion was 5.6 ± 1.6, which included L1-S1 (12), L1-L5 (13), T12-S1 (14), L2-S1 (15), T10-S1 (16), T11-S1 (11), T12-L5 (10), T10-L5 (8), T11-L5 (8), L2-L5 (6), T9-S1 (2), T8-L3 (2), T9-ilium (1), T12-ilium (1), L1-ilium (1), T10-L2 (1), T10-L4 (1), T11-L3 (1), T11-L4 (1), T7-S1 (1), T8-S1 (1), T9-L4 (1), and T9-L5 (1). The average level of decompression was 3.2 ± 1.2. The average operative time was 269.3 ± 63.2 min. The mean blood loss during operation was 1209.1 ± 756.4 ml. The average hospital stay was 12.7 ± 6.4 days.

### Sagittal parameters and clinical outcome

According to Table 1, immediate improvement in sagittal and coronal deformity was found in patients after corrective surgery. The whole sagittal balance and pelvic compensation improved soon after surgery with the reconstruction of LL, and TK also increased. From the time at soon after surgery to the last follow-up, SVA increased 150% (27.6 mm), PT increased 30% (5.4°), TK increased 13% (3.3°), and LL decreased 15% (5.9°). With respect to the clinical outcome, ODI, JOA, and VAS significantly improved at the last follow-up.

**Table 1:** The continuous change in sagittal alignment and clinical symptom

	Preoperative	Soon after surgery	Last follow-up
SVA (mm)	42.8 ± 44.5	18.9 ± 32.9*	46.5 ± 40.6*
PI (°)	48.5 ± 10.8	48.6 ± 10.7	49.0 ± 10.6
PT (°)	23.6 ± 10.6	16.9 ± 9.0*	22.3 ± 9.2*
SS (°)	24.8 ± 10.5	31.6 ± 8.7*	26.7 ± 10.1*
LL (°)	27.4 ± 17.2	39.6 ± 10.0*	33.7 ± 13.3*
TK (°)	21.7 ± 14.6	25.8 ± 10.9*	29.1 ± 12.8*
Cobb (°)	27.7 ± 11.6	9.9 ± 6.4*	10.0 ± 5.8
PI-LL (°)	21.1 ± 17.2	9.0 ± 11.2*	15.3 ± 14.1*
LL-TK (°)	5.7 ± 14.8	13.8 ± 12.4*	4.6 ± 13.6*
ODI	55.4 ± 13.5	–	28.2 ± 18.0*
JOA	13.7 ± 4.6	–	21.5 ± 4.9*
VAS back	5.7 ± 1.8	–	3.1 ± 2.0*
VAS leg	4.7 ± 2.6	–	2.3 ± 1.9*

“*” means that compared with the former stage, *P* < 0.05

In Table 2, we found that preoperative SVA, LL, and LL-TK significantly correlated with preoperative ODI. In Table 3, we could see that SVA, PT, and PI-LL were all significantly associated with postoperative ODI, but among all the parameters, LL-TK showed the strongest correlations with ODI (*r* = − 0.300, *P* < 0.01) and JOA (*r* = 0.361, *P* < 0.01). Meanwhile, we also calculated the relationship between LL-TK at soon after surgery and SVA at the last follow-up (*r* = − 0.185, *P* < 0.05), which demonstrated that LL-TK could also predict the postoperative whole sagittal balance.

**Table 2:** The correlation between preoperative sagittal parameters and health-related quality of life

	SVA	PT	LL	PI-LL	LL-TK	Cobb
ODI	0.287**	0.117	− 0.264**	0.213*	− 0.211*	0.117
JOA	− 0.171	− 0.034	0.148	− 0.107	0.145	− 0.036

“*” means that *P* < 0.05 and “**” means that *P* < 0.01

**Table 3:** The correlation between the sagittal parameters at soon after surgery and health-related quality of life

	SVA	PT	LL	PI-LL	LL-TK	Cobb
ODI	0.188*	0.177*	− 0.098	0.208*	− 0.300**	0.117
JOA	− 0.173*	− 0.158	0.080	− 0.138	0.316**	− 0.134

“*” means that *P* < 0.05 and “**” means that *P* < 0.01

**Fig. 2:**
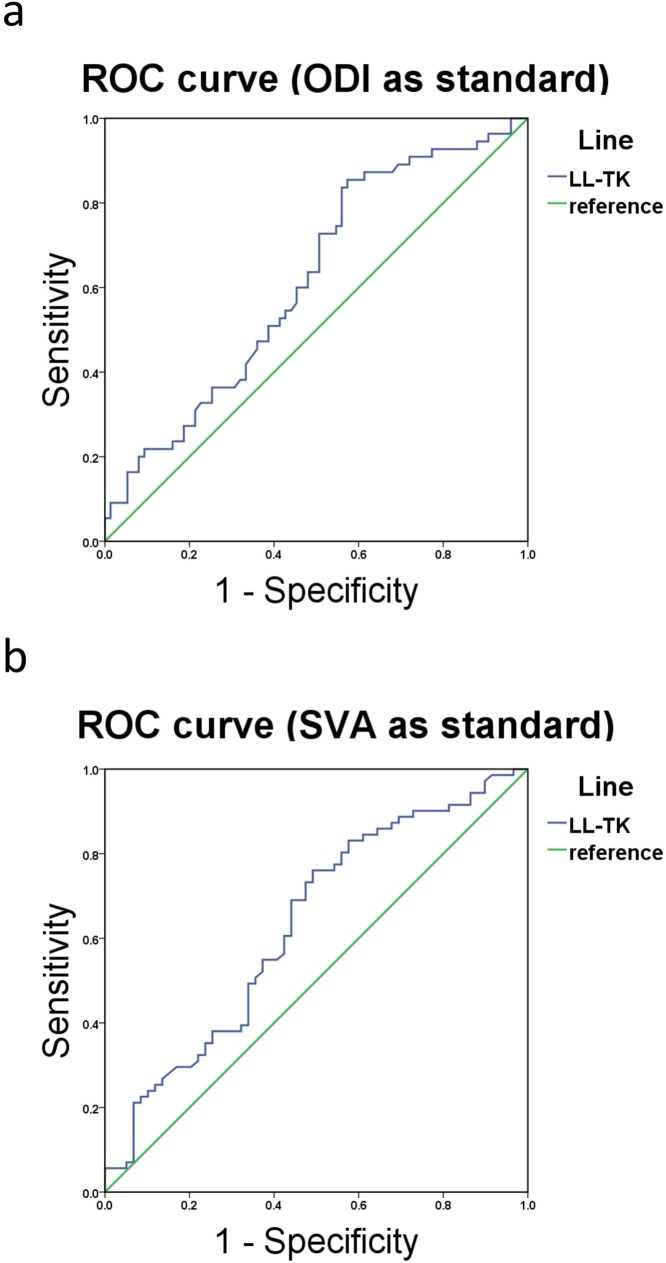
ROC curve to find the optimum cut-off point of LL-TK to predict the clinical outcomes. **a** represents the ROC curve of LL-TK in predicting postoperative ODI. **b** represents the ROC curve of LL-TK in predicting postoperative SVA

To further evaluate the effectiveness of LL-TK in predicting the postoperative clinical outcome and sagittal balance, we used ROC curves and calculated the Youden index (sensitivity plus specificity minus one) to figure out the best cut-off point. We selected ODI as the direct evaluation index for clinical outcomes and divided the patients into ODI > 20 group and ODI ≤ 20 group to distinct the extent of symptom severity (Fig. 2a). Similarly, the patients were divided into sagittal balance group (SVA < 50 mm) and sagittal imbalance group (SVA ≤ 50 mm) to paint the ROC curve (Fig. 2b). In the end, we found that LL-TK = 10° could be used as the best cut-off value accounting for both clinical outcome (AUC = 0.617, sensitivity = 0.764, specificity = 0.440) and sagittal balance (AUC = 0.631, sensitivity = 0.761, specificity = 0.492).

Based on LL-TK = 10°, we divided the patients into two groups to compare their clinical outcome and sagittal parameters. As shown in Table 3, the follow-up time, preoperative situation, and sagittal balance in the LL-TK > 10 group and LL-TK ≤ 10 group had no significant difference (*P* > 0.05), so the two groups were comparable. At the last follow-up, the patients with LL-TK > 10° showed significantly better clinical outcome including lesser ODI (*P* = 0.005), VAS back (*P* = 0.007), and larger JOA (*P* = 0.011) than those with LL-TK ≤ 10°. Meanwhile, there were more patients with sagittal imbalance in LL-TK ≤ 10° group than the other group at the last follow-up. However, no significant difference in the occurrence of proximal junctional kyphosis (PJK) and loosening of fixation was found between the two groups.

## Discussion

Since the sagittal balance was reported to be significantly related with patient’s health-related quality of life (HRQOL) [12–14], the importance of sagittal alignment and sagittal balance has been gradually recognized. A suitable sagittal alignment could save the energy of maintaining erect position and reduce the load of muscle. Many spinal degenerative diseases were accompanied by a decrease in LL and increase in PT, which could usually lead to sagittal imbalance, especially in patients with ADS whose compensatory mechanisms failed to compensate for primary deformity [15].

To rebalance the forward moving center of gravity, the compensatory mechanism involved in the spine (decrease in TK and extension of adjacent segments), pelvis (pelvic retroversion, in other words, increase in PT), and lower extremities (flexion of knee and extension of ankle). As proposed by previous study, LL-TK accounted for the local compensatory mechanism, and LL-TK > 0° could predict a good sagittal balance in elderly population since TK could decrease to compensate the loss in LL [11]. In the long-fixed spine of patients after corrective surgery, the fused sagittal alignment, including lumbar and thoracic curve, as well as pelvic rotation, limited the regional compensatory mechanism: decreased thoracic kyphosis and increased pelvic tilt. So it was still unclear whether the regional parameters expressing as LL-TK could effectively predict the postoperative sagittal balance and HRQOL in patients with ADS.

In the present study, we evaluated the relationships between postoperative sagittal parameters and HRQOL and found that SVA, PT, PI-LL, and LL-TK were all significantly related with postoperative ODI. This result was consistent with previous studies [5, 16, 17], which demonstrated that the sagittal balance, rather than coronal balance, influenced patients’ HRQOL. Among these parameters, LL-TK reflecting the regional balance of lumbar and thoracic spine most highly correlated with ODI (*r* = − 0.300), and it was also associated with SVA (*r* = − 0.185). These results indicated that LL-TK could predict the postoperative HRQOL and sagittal balance. In the normal spine, TK could decrease to compensate the loss of LL and rebalance the spine and LL also increased with increased TK, so LL-TK could remain > 0°. However, this compensatory mechanism was limited in the spine of patient after correction surgery, so the decrease in LL or increase in TK could more directly influence LL-TK, then the sagittal balance. Therefore, the postoperative LL-TK could effectively predict the postoperative sagittal balance and HRQOL.

In addition, by using the ROC curve, we calculated the cut-off value of LL-TK at 10° to reach a good clinical outcome (ODI < 20) and sagittal balance (SVA < 50 mm). Then, we divided the patients into two groups based on LL-TK assessed soon after surgery, and compared the preoperative and postoperative data of the two groups. According to Table 4, group 1 (LL-TK > 10°) showed significantly better VAS back, ODI, and JOA at the last follow-up than group 2 (LL-TK < 10°), and there were more patients with sagittal imbalance in group 2 than group 1. These results demonstrated that LL-TK > 10° could more successfully predict a better clinical outcome in patients with ADS. With respect to the predictive effect, LL-TK > 10° rather than LL-TK > 0° might be more effective, since LL decreased and TK increased, corresponding to decrease in LL-TK from soon after surgery to the last follow-up as shown in Table 1. Interestingly, the decrease in LL-TK was approximately 10°. Lafage et al. [18] defined the ideal threshold of LL-TK ranging from 27.6° for ASD patients aged < 35 years to 2.1° for patients aged > 75 years. Considering the average age (63.6 years) of our patients’ group, our conclusion was generally consistent with their study.

**Table 4:** Comparison between two groups with different values of lumbar lordosis minus thoracic kyphosis at soon after surgery

	LL-TK^2^ > 10	LL-TK^2^ ≤ 10	*P* value
N	84	46	–
Operative time (min)	267.9 ± 69.9	271.8 ± 49.2	0.713
Blood loss (ml)	1220.4 ± 835.6	1188.5 ± 593.0	0.801
Hospital stay (day)	11.9 ± 4.4	14.1 ± 8.7	0.059
Follow-up time (month)	37.3 ± 16.3	40.8 ± 16.1	0.240
SVA^1^ (mm)	37.6 ± 42.6	52.4 ± 46.8	0.079
SVA^2^ (mm)	16.8 ± 32.9	23.6 ± 32.6	0.226
SVA^3^ (mm)	41.7 ± 58.2	58.2 ± 36.3	0.021*
Sagittal imbalance^1^ (N)	29	22	0.137
Sagittal imbalance^2^ (N)	10	10	0.137
Sagittal imbalance^3^ (N)	30	29	0.003**
VAS back^1^	5.6 ± 1.8	5.9 ± 1.7	0.442
VAS back^3^	2.8 ± 1.8	3.9 ± 2.4	0.007**
ODI^1^	54.8 ± 13.5	56.4 ± 13.4	0.529
ODI^3^	24.8 ± 16.1	34.9 ± 20.7	0.005**
JOA^1^	13.6 ± 4.6	13.7 ± 4.7	0.960
JOA^3^	22.3 ± 4.2	19.7 ± 6.1	0.011*
PJK	17	13	0.299
Loosening of fixation	50	31	0.376

“*” means *P* < 0.05. “**” means *P* < 0.01

“^1^” means preoperative. “^2^” means soon after surgery. “^3^” means last follow-up

There were still some limitations in the present study. First, this was only a single-center retrospective study which might bring the selection bias. Second, the sample size was relatively small. Therefore, a multicenter prospective study was needed to further test the effectiveness of LL-TK in predicting the outcome of correction surgery. Third, thoracic kyphosis was not easily determined without instrumentation of all thoracic vertebras. However, the lower thoracic vertebrae account for the major part of thoracic kyphosis, so TK could be controlled greatly in the correction surgery (19). Despite these limitations, in patients with ADS (elderly population), TK was larger and took more part in the condition of sagittal balance, so it should be considered when making correction strategy for these patients apart from the classical parameters, and this study proposed that LL-TK was a good predictor for clinical outcome for patients with ADS, and LL-TK > 10° could be an optimal corrective target.

## Conclusions

LL-TK measured soon after surgery was strongly associated with patients’ HRQOL and SVA at last follow-up, so it was suitable for predicting the surgical outcome for patients after correction surgery. Based on ROC curve, the cutoff value of LL-TK to determine a good clinical outcome (ODI < 20) and sagittal balance (SVA < 50 mm) was set at 10°. Patients with LL-TK > 10° showed significantly better clinical outcome than patients with LL-TK < 10°, so LL-TK > 10° could be a suitable correction target for patients with adult degenerative scoliosis.

## Data Availability

The datasets used and analyzed during the current study are available from the corresponding author on reasonable request.

## Abbreviations

ADS: Adult degenerative scoliosis
ASD: Adult spinal deformity
HRQOL: Health-related quality of life
JOA: Japanese Orthopaedic Association
LL: Lumbar lordosis
ODI: Oswestry Disability Index
PI: Pelvic tilt
SS: Sacral slope
SVA: Sagittal vertical axis
TK: Thoracic kyphosis
VAS: Visual Analog Scale
